# Risk factors of atherosclerotic tissue types in single-vessel and intermediate coronary lesions: a cross-sectional study

**DOI:** 10.1186/s12944-017-0456-z

**Published:** 2017-03-23

**Authors:** Xianjin Wang, Qun Chen, Yu Xu, Yanqing Wang, Yang Yang, Ming Gu, Haihua Xu, Yanfang Zhao

**Affiliations:** 1 Department of Cardiology, the Eighty-first Hospital of PLA Affiliated with Anhui Medical University, No. 34, Biao 34, Yanggongjing, Qinhuai District, Nanjing, Jiangsu 210000 China; 2 Department of Cardiology, the Eighty-first Hospital of PLA Affiliated with the Nanjing University of Chinese Medicine, Nanjing, China

**Keywords:** Arteriosclerosis, Ultrasonics, Lipoprotein-associated phospholipase A2

## Abstract

**Background:**

Few data exist that correlate lesion-related risk factors such as conventional cardiovascular risks or lipoprotein-associated phospholipase A2 (Lp-PLA2) with tissue types within atherosclerotic plaques in patients with single-vessel and intermediate coronary lesions.

**Methods:**

One hundred and ninety-two patients with single-vessel and intermediate coronary lesions were enrolled in a cross-sectional study and divided into two groups: stable angina pectoris (SAP) and acute coronary syndrome (ACS) groups. Data regarding clinical characteristics and Lp-PLA2 mass were collected. Using iMAP-IVUS, lumen areas were manually traced to determine the minimum lumen area (MLA) at 1-mm intervals in diseased segments. At the minimum lumen lesion, areas of different types of atherosclerotic tissue [i.e., areas of fibrous plaque tissue (FP), fibro-fatty tissue (FF), dense calcium (DC) and necrotic core (NC)], vascular external elastic membrane (EEMCSA) and plaque and media (P&MCSA) were calculated using the built-in iMap algorithm. Plaque burden was computed as P&MCSA divided by EEMCSA.

**Results:**

In a univariate analysis, glycosylated hemoglobin A1C (GHbA1C), low-density lipoprotein cholesterol (LDL-C), high-density lipoprotein cholesterol (HDL-C), hypertension, Lp-PLA2 and a history of taking statins predicted the degree of FP and NC area, as well as plaque burden, but were not significant predictors of FF or DC area. In a multivariate analysis, Lp-PLA2 and HbA1c remained independent predictors of plaque burden, FP and NC area. However, the results of the regression analyses were not identical when the SAP and ACS groups were analyzed separately. Lp-PLA2, diabetes and NC area were significant predictors of acute coronary lesions, and the predictive value of Lp-PLA2 was confirmed by the finding of a high area-under-the-curve in a ROC analysis (0.837, 95% CI:0.778-0.895, *P =* 0.000), as well as by the reasonable sensitivity and specificity of cut-off values.

**Conclusions:**

GHbA1C and Lp-PLA2 were strong independent predictors of plaque burden and FP and NC area at the minimum lumen lesion in patients with single-vessel and intermediate coronary lesions. Furthermore, Lp-PLA2 has a certain predictive value for acute coronary lesions.

## Background

Vulnerable atherosclerotic plaques, which are associated positively with the presence of more unstable atherosclerotic tissue leading to adverse cardiovascular events, often occur at sites of angiographically intermediate coronary-artery stenosis. Atherosclerosis and thrombosis are markedly pathogenic mechanisms that are associated with the majority of cardiovascular events. Epidemiologic data show that the relationship between conventional cardiovascular risk factors and adverse clinical events in patients with coronary disease is complex [1]. Lp-PLA2 was introduced as an underlying important pathogenic factor that participates in the generation of pro-atherogenic metabolites, such as oxidized free fatty acids and lysophosphatidylcholine [2]. Elevated Lp-PLA2 activity and plasma levels promote an increased risk of coronary events [3]. Numerous previous reports have shown that the predictors of culprit or non-culprit lesion-related major adverse cardiovascular events are associated with a combination of thin-cap fibroatheroma (TCFA), plaque burden ≥70%, MLA ≤4.0 mm^2^, median DC area ≥0.2 mm^2^, and median NC area ≥0.4 mm^2^ [4–6]. Whether established cardiovascular risk factors or Lp-PLA2 can predict different tissue types within the atherosclerotic plaque, however, remains unknown. The purpose of this study was to examine the relationship between conventional cardiovascular risk factors, Lp-PLA2 concentration and plaque structure parameters as assessed by intravascular ultrasound in patients with either stable angina or acute coronary syndromes due to intermediate single-vessel coronary artery lesions.

## Methods

### Patients

One hundred and ninety-two patients admitted to the Eighty-first Hospital of PLA (Nanjing, China) were enrolled in a cross-sectional study according to the following criteria: a “target vessel” with angiographically single-vessel and intermediate coronary lesions (40–70% diameter stenosis by visual estimation) subjected to IVUS examination; the case was considered eligible only if the artery had never undergone revascularization with previous percutaneous coronary intervention or coronary-artery bypass grafting. Patients with multi-vessel coronary artery disease were removed. The enrolled patients were divided into two groups: SAP (104 cases) and ACS (88 cases with 10.2% non-STEMI and 89.8% unstable angina) groups. A history of more than one year with diabetes, hypertension or smoking (five or more cigarettes per day) and more than one month of taking aspirin or clopidogrel, statins, angiotensin-converting enzyme inhibitor or angiotensin receptor antagonist (ACEI/ARB) were recorded as a distinction for use as a categorical variable. ACS was defined as unstable angina or myocardial infarction with or without ST-segment elevation. Written informed consent was obtained from each patient before initiating the coronary arteriography and IVUS procedure. The protocol was approved by the Ethics Committee of the Eighty-first Hospital of PLA Affiliated to Nanjing University of Chinese Medicine (Nanjing, China). One or more stents was implanted in some of the patients according to the diameter of MLA (≤0.4 mm^2^) and the length of the lesion-containing vessel.

### Laboratory measurements

All data regarding cardiovascular risk factors and Lp-PLA2 mass were measured before initiating the coronary arteriography and IVUS procedure. Fasting venous blood was collected after admission to the hospital using a vacuum blood collection tube coated with EDTA. After mixing for 10 to 20 min, the plasma samples were separated by centrifugation at 1000 to 2000 revolutions per minute for 20 min at 4 °C. All laboratory examinations were performed in series from aliquots stored at −80 °C using commercial kits; the tests included the following: total cholesterol and triglycerides were measured using an ARCHITECT c800 analyzer (Abbott Laboratories, Germany), HDL-C were measured using DOT Diagnostics kits (Czech Republic). LDL was calculated using the Friedewald equation; i.e., LDL = Total cholesterol − HDL-C − (triglycerides / 2.22). HbA1c was estimated based on high-performance liquid chromatography using a G7 analyzer (TOSOH, Japan).

Lp-PLA2 mass was determined using a commercial Lp-PLA2-ELISA kit (the PLAC test, supplied by NRM Inc., Nanjing, China), which is a sandwich enzyme immunoassay kit that uses two highly specific monoclonal antibodies for the measurement of Lp-PLA2 concentration. A set of calibrators was used to plot a standard curve of absorbance versus Lp-PLA2 concentration; the Lp-PLA2 concentration in the test sample was determined based on this curve, and this ELISA assay had a detection limit of 100 μg/l. All plasma samples were tested in duplicate. The intra-assay coefficient of variation was less than 3.8%, and the inter-assay coefficient of variation was less than 4.5%.

### IVUS disease burden

Quantitative IVUS analysis was performed using the built-in iMap planimetry function in iMap-IVUS (Boston Scientific, Natick, MA, USA). Initially, areas of lumen and vessel inside an external elastic membrane were manually traced to determine the MLA at every 1-mm interval in diseased segments. Thereafter, areas of different types of atherosclerotic tissue were calculated at the most severe stegnotic lesion. Cross-sectional areas of MLA, P&MCSA and EEMCSA were calculated simultaneously. P&MCSA = EEMCSA − MLA, and plaque burden = P&MCSA /EEMCSA *100%. All iMAP data were analyzed via standardized criteria at a central core laboratory.

### Statistical analysis

Statistical analysis was performed using SPSS software (Version 19.0 IBM, Armonk, NY, USA). Categorical variables are presented as frequencies or percentages and were compared using chi-square statistics or the Fisher exact test. Continuous variables were tested for normal distribution via the non-parametric one-sample Kolmogorov–Smirnov test, are presented as the mean ± SD, and were compared using the independent samples *t*-test. Univariate predictors were obtained using linear regression analysis. Multivariate analysis involved multiple linear regression analysis with backward or stepwise selections to determine the risk factors for dependent variables, and only variables with p ≤0.1 based on univariate analysis were included. Logistical regression was used to determine the risk factors for ACS, and a ROC curve was used to describe the diagnostic value. A value of *p <*0.05 was considered statistically significant.

## Results

### Baseline data of the two groups

The clinical characteristics are shown in Table 1; based on these, we can conclude that compared to the SAP group, the ACS group had higher population proportions with histories of smoking and diabetes, higher LDL-C values and Lp-PLA2 masses, and lower HDL-C values. All patients had a history of taking aspirin or clopidogrel for at least one month. Lesion-related coronaries and IVUS disease burden are summarized in Table 2. The values of EEMCSA, P&MCSA, plaque burden, and areas of FP and NC were all higher in the ACS group.

**Table 1:** Patient demographics and laboratory parameters

Variables	SAP (*n =* 104)	ACS (*n =* 88)	*p* Value
Male gender	57(54.81)	59(67.05)	0.085
Hypertension	66(63.46)	54(61.36)	0.765
Diabetes	24(23.08)	48(54.55)	0.000
Smoker	30(28.85)	38(43.18)	0.039
Age, years	65.78 ± 11.51	67.74 ± 12.18	0.254
GHbA1C, mg/dl	6.22 ± 1.00	7.08 ± 1.57	0.000
Lp-PLA2, μg/l	188.24 ± 58.95	271.40 ± 58.53	0.000
Triglycerides, mmol/l	1.66 ± 1.39	1.98 ± 1.48	0.125
Total cholesterol, mmol/l	4.26 ± 1.10	4.58 ± 1.28	0.062
LDL-C, mmol/l	2.68 ± 0.97	3.12 ± 1.14	0.004
HDL-C, mmol/1	1.12 ± 0.27	0.99 ± 0.29	0.003
Medication			
Aspirin/ clopidogrel	104(100)	88(100)	1.000
ACEI/ARB	53(50.96)	40(45.45)	0.447
Statins	81(77.88)	62(70.45)	0.239

**Table 2:** Lesion-related coronaries and IVUS characteristics

Variables	SAP (*n =* 104)	ACS (*n =* 88)	*p* Value
Lesion-bearing vessel			
Left anterior descending (LAD)	51(49.14)	49(55.68)	0.359
Right coronary artery (RCA)	32(30.77)	24(27.27)	0.595
Left circumflex (LCX)	21(20.19)	15(17.05)	0.578
IVUS data			
External elastic membrane (EEMCSA), mm^2^	11.21 ± 2.44	12.54 ± 2.55	0.000
Minimum lumen area (MLA), mm^2^	4.30 ± 1.42	4.00 ± 1.30	0.133
Plaque and media (P&MCSA), mm^2^	6.91 ± 1.86	8.54 ± 2.12	0.000
Plaque burden, %	61.58 ± 9.13	67.77 ± 8.29	0.000
Fibrous plaque tissue (FP), mm^2^	5.42 ± 1.66	6.46 ± 2.04	0.000
Fibro-fatty tissue (FF), mm^2^	0.54 ± 0.48	0.53 ± 0.31	0.927
Necrotic core (NC), mm^2^	0.66 ± 0.32	1.25 ± 0.53	0.000
Dense calcium (DC), mm^2^	0.29 ± 0.23	0.29 ± 0.21	0.958
Stent implantation	50(48.08)	47(53.41)	0.463

### Univariate predictors of IVUS disease burden

The univariate analysis predictors of atherosclerotic tissue types and plaque burden are summarized in Table 3 (placed at the end of the document text file because the table takes up more than one page). Regarding the area of FP and plaque burden, GHbA1C, total cholesterol, triglycerides, LDL-C, and Lp-PLA2 were found to be predictors of more severe disease, and HDL-C and statins were found to be protective factors. Regarding the area of NC, GHbA1C, total cholesterol, LDL-C, age, hypertension, and Lp-PLA2 were found to be predictors of more severe disease, and HDL-C was found to be a protective factor. There were no significant predictors for areas of tissues of FF and DC.

**Table 3:** Univariate predictors of IVUS disease burden (*n =*192)

Variables	FP area βCoeff.(95% CI)	FF area βCoeff.(95% CI)	NC area βCoeff.(95% CI)	DC area βCoeff.(95% CI)	Plaque burden βCoeff.(95% CI)
Male gender	0.245 (−0.313 to 0.802)	0.023 (−0.097 to 0.144)	0.062 (−0.091 to 0.214)	−0.008 (−0.073 to 0.056)	0.668 (−2.034 to 3.370)
Hypertension	0.205 (−0.359 to 0.768)	−0.091 (−0.212 to 0.030)	0.167** (0.015 to 0.320)	0.034 (−0.031 to 0.100)	0.467 (−2.264 to 3.197)
Smoker	0.340 (−0.230 to 0.909)	−0.053 (−0.176 to 0.070)	0.137* (−0.018 to 0.292)	−0.025 (−0.091 to 0.041)	0.835 (−1.927 to 3.597)
Age	0.004 (−0.019 to 0.027)	0.0004 (−0.005 to 0.005)	0.007** (0.001 to 0.013)	0.001 (−0.001 to 0.004)	0.045 (−0.067 to 0.157)
GHbA1C	0.688** (0.511 to 0.864)	0.015 (−0.028 to 0.059)	0.098** (0.045 to 0.151)	0.012 (−0.012 to 0.035)	3.149** (2.282 to 4.017)
Triglycerides	0.330** (0.144 to 0.515)	0.009 (−0.032 to 0.051)	0.047* (−0.004 to 0.099)	−0.011 (−0.033 to 0.011)	1.412** (0.509 to 2.315)
Total cholesterol	0.508** (0.290 to 0.726)	0.017 (−0.032 to 0.067)	0.067** (0.005 to 0.129)	−0.013 (−0.039 to 0.014)	1.394** (0.299 to 2.489)
LDL-C	0.689** (0.454 to 0.925)	0.018 (−0.037 to 0.073)	0.092** (0.024 to 0.161)	−0.015 (−0.045 to 0.014)	2.009** (0.808 to 3.120)
HDL-C	−1.366** (−2.293 to −0.440)	−0.135 (−0.339 to 0.086)	−0.320** (−0.574 to −0.065)	−0.010 (−0.120 to 0.100)	−9.266** (−13.654 to −4.879)
ACEI/ARB	0.133 (−0.413 to 0.679)	−0.109* (−0.226 to 008)	0.014 (−0.136 to 0.163)	−0.003 (−0.067 to 0.060)	−1.350 (−3.989 to 1.289)
Statins	−0.843** (−1.458 to −0.228)	−0.042 (−0.177 to 0.093)	−0.043 (−0.214 to 0.128)	0.046 (−0.026 to 0.119)	−3.140** (−6.139 to −0.140)
Lp-PLA2	0.007** (0.004 to 0.011)	0.000 (0.000 to 0.001)	0.004** (0.003 to 0.005)	0.000 (−0.001 to 0.000)	0.038** (0.020 to 0.056)

*FP* fibrous plaque tissue, *FF* fibro-fatty tissue, *NC* necrotic core, *DC* dense calcium

*p <0.10

**p <0.05

### Multivariate predictors of IVUS disease burden

The multivariate analysis predictors are summarized in Table 4. In the multivariate analysis, after adjustment for other risk factors, both Lp-PLA2 and GHbA1C remained independent predictors of more severe disease for plaque burden as well as areas of FP and NC. HDL-C remained a protective factor either for plaque burden or area of FP in agreement with the univariate regression models. Suffering from hypertension was independently associated with increased area of NC.

**Table 4:** Multivariate predictors of IVUS disease burden (*n =*192)

Variables	Selection = Backward^a^ βCoeff.(95% CI)	*p* Value	Selection = Stepwise^a^ βCoeff.(95% CI)	*p* Value
Plaque burden				
GHbA1C	2.540(1.677 to 3.404)	0.000	2.712(1.863 to 3.561)	0.000
Total cholesterol	1.135(−0.078 to 2.349)	0.066		
HDL-C	−7.366(−11.688 to −3.043)	0.001	−5.736(−9.718 to −1.755)	0.005
Statins	−2.227(−5.296 to 0.843)	0.154	−3.860(−6.401 to −1.319)	0.003
Lp-PLA2	0.020(0.004 to 0.036)	0.015	0.022(0.006 to 0.038)	0.006
FP area				
GHbA1C	0.562(0.391 to 0.733)	0.000	0.576(0.408 to 0.744)	0.000
LDL-C	0.521(0.268 to 0.775)	0.000	0.613(0.405 to 0.821)	0.000
HDL-C	−0.833(−1.626 to −0.040)	0.040	−0.997(−1.780 to −0.214)	0.013
Statins	−0.273(−0.881 to 0.335)	0.376		
Lp-PLA2	0.003(0.000 to 0.006)	0.044		
NC area				
Hypertension	0.163(0.043 to 0.283)	0.008	0.162(0.041 to 0.282)	0.009
GHbA1C	0.042(−0.003 to 0.088)	0.065	0.052(0.007 to 0.096)	0.022
HDL-C	−0.147(−0.372 to 0.077)	0.197		
Total cholesterol	0.052(−0.001 to −0.105)	0.055		
Lp-PLA2	0.004(0.003 to 005)	0.000	0.004(0.003 to 0.005)	0.000

^a^Variables selected are based on a significance level set at a *p* value ≤0.1 via univariate analysis

### Univariate and multivariate predictors of IVUS disease burden in SAP and ACS groups

The results of the regression analyses were not identical when the SAP and ACS groups were analyzed separately, whether using a univariate analysis or a multivariate analysis, as shown in the following Tables 5 and 6. Compared to the ACS group, in addition to GHbA1C and LDL-C, Lp-PLA2 and a history of taking statins were found to be predictors of FP area in the SAP group. A history of taking ACEI/ARB was a predictor of FP area in the SAP group but not in the ACS group. Hypertension and Lp-PLA2 were predictors of NC area in both groups. The predictors of DC area were taking statins in the SAP group and male gender in the ACS group. The predictors of plaque burden were HDL-C in the SAP group and LDL-C in the ACS group, and GHbA1C was a predictor in both groups.

**Table 5:** Univariate and multivariate predictors of IVUS disease burden in the SAP group

	FP area		FF area		NC area		DC area		Plaque burden	
	Univariate	Multivariate	Univariate	Multivariate	Univariate	Multivariate	Univariate	Multivariate	Univariate	Multivariate
	*P* Value	*P* Value	*P* Value	*P* Value	*P* Value	*P* Value	*P* Value	*P* Value	*P* Value	*P* Value
Male gender	0.858		0.137		0.993		0.186		0.985	
Hypertension	0.106		0.042		0.047	0.039	0.916		0.792	
Smoker	0.521		0.681		0.885		0.913		0.931	
Age	0.447		0.382		0.066		0.919		0.984	
GHbA1C	0.000	0.000	0.988		0.043		0.198		0.000	0.001
Triglycerides	0.059		0.891		0.655		0.143		0.061	
Total cholesterol	0.000		0.926		0.502		0.439		0.408	
LDL-C	0.000	0.054	0.983		0.378		0.555		0.148	
HDL-C	0.040		0.293		0.160		0.464		0.002	0.005^a^
ACEI/ARB	0.729		0.039	0.039^a^	0.156		0.400		0.487	
Statins	0.000	0.002^a^	0.892		0.975		0.018	0.018	0.218	
Lp-PLA2	0.009	0.033	0.163		0.000	0.000	0.067		0.038	

^a^The β coefficient was found to be negative in the multivariate analysis (with stepwise selection)

**Table 6:** Univariate and multivariate predictors of IVUS disease burden in the ACS group

	FP area		FF area		NC area		DC area		Plaque burden	
	Univariate	Multivariate	Univariate	Multivariate	Univariate	Multivariate	Univariate	Multivariate	Univariate	Multivariate
	*P* Value	*P* Value	*P* Value	*P* Value	*P* Value	*P* Value	*P* Value	*P* Value	*P* Value	*P* Value
Male gender	0.691		0.064		0.789		0.040	0.040^a^	0.901	
Hypertension	0.755		0.621		0.041	0.034	0.142		0.689	
Smoker	0.768		0.357		0.383		0.229		0.810	
Age	0.509		0.151		0.330		0.181		0.501	
GHbA1C	0.000	0.000	0.221		0.640		0.710		0.000	0.000
Triglycerides	0.013		0.518		0.307		0.839		0.054	
Total cholesterol	0.015		0.250		0.298		0.561		0.044	
LDL-C	0.004	0.003	0.236		0.389		0.375		0.040	0.039
HDL-C	0.268		0.368		0.810		0.608		0.137	
ACEI/ARB	0.521		0.911		0.951		0.409		0.684	
Statins	0.817		0.352		0.804		0.511		0.207	
Lp-PLA2	0.704		0.657		0.000	0.000	0.838		0.743	

^a^ The β coefficient was found to be negative in the multivariate analysis (with stepwise selection)

### Diabetes and IVUS disease burden

Table 7 summarizes the relationships between IVUS disease burden and the presence or absence of diabetes. All IVUS data regarding disease burden were likely to be more severe in the diabetes group, including P&MCSA, EEMCSA, MLA, plaque burden, and areas of FP and NC tissues. However, areas of either FF or DC tissues were not significantly different between patients with or without diabetes.

**Table 7:** IVUS data in patients with diabetes VS patients without diabetes

Variables	Diabetes (*n =* 72)	Non-diabetes (*n =* 120)	*p* Value
EEMCSA, mm^2^	12.74 ± 2.52	11.27 ± 2.45	0.000
MLA, mm^2^	3.88 ± 1.17	4.33 ± 1.45	0.028
P&MCSA, mm^2^	8.86 ± 2.16	6.94 ± 1.78	0.000
Plaque burden, %	69.11 ± 7.73	61.60 ± 8.99	0.000
FP area, mm^2^	6.92 ± 1.92	5.28 ± 1.63	0.000
FF area, mm^2^	0.54 ± 0.37	0.53 ± 0.44	0.890
NC area, mm^2^	1.09 ± 0.51	0.84 ± 0.51	0.001
DC area, mm^2^	0.31 ± 0.23	0.28 ± 0.21	0.465

*EEMCSA* cross-sectional area of external elastic membrane, *MLA* minimum lumen area, *P&MCSA* cross-sectional area of plaque and media

### Risk factors for the incidence of ACS identified by logistic regression

Multivariate models including all factors (i.e., conventional cardiovascular risks, Lp-PLA2, plaque burden and areas of atherosclerotic tissue types) were analyzed to determine the risk factors for the incidence of ACS in patients with single-vessel and intermediate coronary lesions by logistic regression. Forward selection was chosen to determine the relevant variables. This model was based only on the data for the 192 patients enrolled in our study (as summarized in Table 8); based on the results, we concluded that male gender, LDL-C, Lp-PLA2, diabetes and area of NC were independent risk factors.

**Table 8:** Risk factors for the incidence of ACS

Variables	OR value	95% CI	Wald value	P
Male gender	2.832	1.168 to 6.864	5.309	0.021
Diabetes	3.188	1.406 to 7.228	7.703	0.006
Lp-PLA2	1.015	1.008 to 1.022	17.720	0.000
LDL-C	1.687	1.112 to 2.559	6.047	0.014
NC area	9.155	3.102 to 27.015	16.085	0.000

### ROC curves for the predictors of the incidence of ACS

**Fig. 1:**
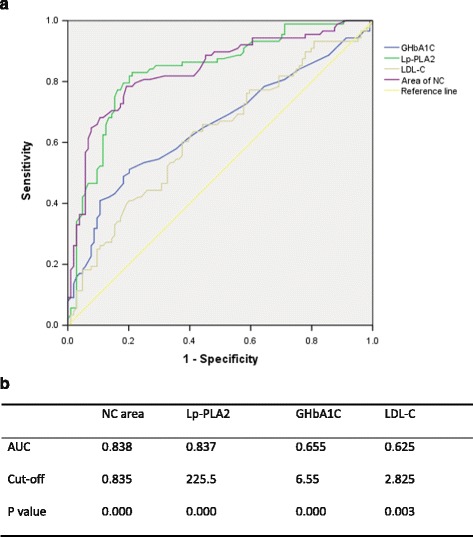
Predictive value of Lp-PLA2, GHbA1C, LDL-C and area of NC for ACS. **a** ROC curves for the four variables. **b** Area under the curve (AUC) and cut-off values

Lp-PLA2, GHbA1C (representing diabetes), LDL-C and NC area were selected as strong risk factors for ACS by logistic regression as mentioned above. ROC curves were used to evaluate their diagnostic value and are described in Fig. 1 (provided as a separate file named “Fig. 1”); we found that the area under the ROC curve regarding the Lp-PLA2-predicted incidence of ACS in the 192 patients was 0.83 (95%CI: 0.778–0.895, *P =* 0.000); the cut-off value was 225.5 μg/L, providing a sensitivity of 76.84% and a specificity of 84.54%. In contrast, GHbA1c and LDL-C had lower predictive values.

## Discussion

Markers of coronary atherosclerotic plaque vulnerability, such as a thin fibrous cap, increased inflammatory activity, and a high lipid burden, have been identified in histological studies. Vulnerable atherosclerotic plaques that lead to adverse cardiovascular events often occur in the late stage of coronary artery atherosclerosis and at sites of angiographically mild or intermediate coronary-artery stenosis, from which we might argue that angiography of the coronary artery does not describe the true extent of atherosclerosis but only luminal narrowing. Grayscale IVUS has been used frequently in vivo as a high-resolution technique for the quantitative assessment of coronary vessel wall anatomy and to provide more in-depth information than conventional angiography but is unable to precisely distinguish between noncalcific tissue types within the plaque. To solve this problem, novel modalities of intravascular imaging have been developed to search for vulnerable plaques by directly measuring various tissue types within the atherosclerotic plaque. In light of this information, the so-called “three stars” of IVUS (virtual histology (VH) IVUS, the integral of backscatter intensities (IB) IVUS, and iMAP-IVUS) have been introduced. These techniques are based on original radio-frequency IVUS signals, which are inherently limited for characterizing tissues, especially in terms of discriminating fatty and fibrous tissues [7, 8]; the signals are reprocessed to identify high-risk atherosclerotic lesions, and each of the techniques uses a specific mathematical model. The iMap-IVUS technique used in the present study is based on a neural network theory and identifies each tissue using a unique concept of “confidence level” [9]. Unfortunately, when using iMAP-IVUS, the number of reference points for lipid areas is smaller than that for other types of atherosclerotic tissue.

Identification of circulating risk or protection markers that contribute to improving the prediction of adverse cardiovascular events is, currently, on the frontiers of cardiology. Two previous studies have suggested that male gender and diabetes are strong predictors of atherosclerotic plaque burden in patients with coronary artery disease, although other risks, such as hypertension, a history of smoking, and prior revascularization or stroke, remain significant [10, 11]. Compared to previous reports [10, 11], the patients reported in this study had relatively mild stenosis (MLA, 4.16 mm^2^ vs. 1.73 mm^2^ and 2.26 mm^2^). In this study, older patients, more patients with diabetes, and more smokers were included, as were fewer males; thus, the differences reported may be due to the differences between the analyzed population segments. Notably, both of the two previous studies lacked data regarding the tissue types within the plaque due to the inherent limitations of grayscale IVUS. In the present study, in addition to diabetes, hyperlipidemia (including higher values of either LDL-C or HDL-C) was associated with plaque burden and area of FP; this finding is partially consistent with a previous report of a strong correlation between the serial accumulation of atheroma and LDL levels [12]. Nevertheless, although LDL-C and HDL-C were found predictive in our univariate analysis, in terms of their association with the area of NC, neither was an independent predictor. In light of these findings, we suggest that lipid levels might be independent of their impact on plaque burden and area of FP tissue; however, we could not rule out the hypothesis regarding their role in determining plaque vulnerability.

In contrast, male gender and a history of smoking were less important than expected in this study; this might have been mainly due to differences in the sample composition and target points. This study included older patients, which may lead to a diminished protective effect of estrogen for females who suffer less coronary heart disease. However, the data regarding the atherosclerotic plaque characteristics investigated in the present study only contained point variables within the most diseased segment, except for the three-dimensional variables, such as average plaque area and percent plaque volume, which may also have affected the risks factor analysis, positively; this was also considered a potential data limitation.

All IVUS data regarding disease burden were likely to have been more severe in diabetes group in this study. We therefore hypothesized that the diabetic state is responsible for the development of atherosclerotic plaque based on relatively convincing evidence, and this evidence suggested a strong correlation between enhanced atheroma burden and increased event rates of ACS. Various abnormalities of hematologic and vascular function in patients with diabetes might result in endothelial dysfunction and subsequently enhanced platelet activity [13]. However, the detailed pathogenic mechanisms correlating plaque tissue types with diabetes have not been extensively investigated.

Currently, LP-PLA2 has been introduced as a new marker of atherosclerotic plaque destabilization, which plays a key role in the generation of pro-atherogenic metabolites and the metabolism of pro-inflammatory phospholipids. In the present study, Lp-PLA2 independently showed significant correlations with the IVUS data regarding disease burden, especially with the area of NC, and exhibited a high predictive value for acute coronary lesions; this result might agree with the results of a trial in dyslipidemic and diabetic pigs: darapladib (which exhibits a pharmacological inhibition of Lp-PLA2) reduced Lp-PLA2 levels in coronary atherosclerotic plaques and decreased the necrotic core in the plaques [14]. However, the results of two randomized trials (STABILITY[15] and the SOLID-TIMI 52 [16], which were conducted to test whether darapladib reduces cardiovascular events in stable and unstable coronary heart disease) did not demonstrate any beneficial effect on any of the primary endpoints (cardiovascular death, stroke, myocardial infarction, or urgent coronary artery revascularization). These disappointing results might seem to cast doubt on the pathogenic role of Lp-PLA2 in atherosclerotic plaque destabilization. However, taking into consideration the well-founded plethora of data demonstrating that Lp-PLA2 predicts cardiovascular events, one could argue that Lp-PLA2 does not play a causative role in these events but only serves as a prognostic marker.

Statins, cholesterol-lowering drugs, were found to reduce plasma Lp-PLA2 activity because most plasma Lp-PLA2 is linked to LDL-C [17, 18]. Treatment with statin brings demonstrable benefits in terms of lowering mortality and cardiovascular event rates, and this might be explained by the reduction of Lp-PLA2, which was demonstrated to be predictive, or even more predictive, than a decrease in LDL cholesterol [17]. In this study, a history of taking statins was found to be a predictor of FP area only in the SAP group (not in the ACS group) after adjusting for other factors, including LDL-C and Lp-PLA2. However, none of these studies was able to determine whether the decrease of Lp-PLA2 was associated with a reduction of the associated enzyme activity in the atherosclerotic plaque.

Several potential limitations of our study should be noted. First, this study was based on a relatively small sample. Second, based on this single-center and cross-sectional study, we could not investigate more deeply regarding the correlations between conventional cardiovascular risk factors, Lp-PLA2 and cardiovascular events due to the lack of follow-up data.

## Conclusions

The data obtained in the present study indicate that GHbA1C and Lp-PLA2 are strong independent predictors of plaque burden and areas of FP and NC in the most severe stegnotic lesions in patients with single-vessel and intermediate coronary lesions. LDL-C and HDL-C were found to be independent predictors of plaque burden and the area of FP but not for the area of NC. However, hypertension was independently associated with an increased area of NC. No significant predictors were found for areas of tissues of FF and DC mainly due to limitations involving the discrimination of the two tissues when using iMAP-IVUS. Lp-PLA2 has a certain predictive value for the incidence of ACS in patients with single-vessel and intermediate coronary lesions. Future studies are warranted to evaluate whether decreasing the mass of Lp-PLA2 using an effective antagonist will abate the areas of plaque tissue, especially NC tissue, thus reducing the incidence of cardiovascular events.

## Abbreviations

ACS: Acute coronary syndrome
DC: Dense calcium
EEMCSA: Cross-sectional area of external elastic membrane
FF: Fibro-fatty tissue
FP: Fibrous plaque tissue
GHbA1C: Glycosylated hemoglobin A1C
HDL-C: High-density lipoprotein cholesterol
IVUS: Intravascular ultrasound
LDL-C: Low-density lipoprotein cholesterol
Lp-PLA2: Lipoprotein-associated phospholipase A2
MLA: Minimum lumen area
NC: Necrotic core
P&MCSA: Cross-sectional area of plaque and media
SAP: Stable angina pectoris
